# Oncogenic *HSP60* regulates mitochondrial oxidative phosphorylation to support Erk1/2 activation during pancreatic cancer cell growth

**DOI:** 10.1038/s41419-017-0196-z

**Published:** 2018-02-07

**Authors:** Chao Zhou, Hongwei Sun, Chen Zheng, Jing Gao, Qingzi Fu, Nianqi Hu, Xiaoli Shao, Yingying Zhou, Jingting Xiong, Ke Nie, Huaibin Zhou, Lijun Shen, Hezhi Fang, Jianxin Lyu

**Affiliations:** 1 Key Laboratory of Laboratory Medicine, Ministry of Education, Zhejiang Provincial Key Laboratory of Medical Genetics, College of Laboratory Medicine and Life Sciences, Wenzhou Medical University, Wenzhou, Zhejiang China; 2 Department of Clinical Laboratory, Children’s Hospital of Zhejiang University School of Medicine, Hangzhou, China; 3 The First Affiliated Hospital of Wenzhou Medical University, Wenzhou, Zhejiang China; 4 Hangzhou Medical College, Hangzhou, Zhejiang China

## Abstract

HSP60 is a mitochondrial localized quality control protein responsible for maintaining mitochondrial function. Although *HSP60* is considered both a tumor suppressor and promoter in different types of cancer, the role of *HSP60* in human pancreatic ductal adenocarcinoma (PDAC) remains unknown. In this study, we demonstrated that *HSP60* was aberrantly expressed in human pancreatic cancer tissues and cell lines. Analysis of the Cancer Genome Atlas database revealed that *HSP60* expression is positively correlated with pancreatic cancer. Further, knockdown of *HSP60* attenuated pancreatic ductal cancer cell proliferation and migration/invasion, whereas ectopic expression of *HSP60* increased tumorigenesis. Using an in vivo tumorigenicity assay, we confirmed that *HSP60* promoted the growth of pancreatic ductal cancer cells. Functional analyses demonstrated that *HSP60* plays a key role in the regulation of mitochondrial function. Mechanistically, both *HSP60* knockdown and oxidative phosphorylation (OXPHOS) inhibition by metformin decreased Erk1/2 phosphorylation and induced apoptosis and cell cycle arrest, whereas Erk1/2 reactivation with EGF promoted cell proliferation. Intriguingly, in vitro ATP supplementation partially restored Erk1/2 phosphorylation and promoted proliferation in PDAC cells with *HSP60* knockdown and OXPHOS inhibition. These results suggest that mitochondrial ATP is an important sensor of Erk1/2 regulated apoptosis and the cell cycle in PDAC cells. Thus, our findings indicate for the first time that *HSP60* may serve as a novel diagnostic target of human pancreatic cancer, and that inhibition of mitochondrial function using drugs such as metformin may be a beneficial therapeutic strategy targeting pancreatic cancer cells with aberrant function of the HSP60/OXPHOS/Erk1/2 phosphorylation axis.

## Introduction

Mitochondrial functions, particularly oxidative phosphorylation (OXPHOS), are monitored by several hierarchical quality control (QC) machineries^1^. Disturbing of mitochondrial QC proteins have been associated with a number of diseases^2,3^. HSP60 is a mitochondrial matrix localized QC proteins in eukaryote cells. Changes of HSP60 function results in mitochondrial dysfunction and is closely associated with cancer^4^. Inhibition of HSP60 activity with myrtucommulone induces mitochondrial-mediated cancer cell apoptosis. Because HSP60 is a dual regulator of apoptosis, it has been considered both a tumor suppressor and promoter in different cancer types^5,6^.

Pancreatic ductal adenocarcinoma (PDAC) is one of the leading causes of death among all cancers worldwide^7^. Because of its late diagnosis and very poor prognosis, the mortality of pancreatic cancer is almost equal to its incidence. In China, the incidence of pancreatic cancer continually increased from 2000 to 2011^8^. Recently, multiple metabolic reprogramming profiles including the Warburg phenotype, the reverse Warburg phenotype, the glutaminolysis phenotype, and the lipid-dependent phenotype were stratified into different subsets of PDAC cells^9^. Although mitochondria play a central role in the regulation of metabolic flux, aberrant regulation of mitochondrial functions has been associated with PDAC^10^. Sustained OXPHOS function with high-mobility group box 1 (HMGB1) ^11^, the MYC proto-oncogene/ PPARgamma coactivator 1 alpha (PGC-1α) axis^12^, and receptor for advanced glycation endproducts (RAGE) (also known as AGER) have been associated with poor prognosis of PDAC^11^. Despite imbalanced adenosine triphosphate (ATP) generation being central to cancer cell fate decision, the underlying mechanism is not fully understood^11^.

Proteomics analysis has identified several potential protein biomarkers; however, whether there is altered expression of *HSP60* in PDAC and normal tissues is not clear. To explore the mechanisms of QC proteins in PDAC, we performed a bioinformatics analysis of QC transcriptomes and discovered that *HSP60* sustained mitochondrial function driving the development of PDAC. We found that HSP60 regulated the generation of mitochondrial ATP, which is critical for Erk1/2 (a ras-dependent extracellular signal-regulated kinase)  derived anti-apoptotic and cell survival in PDAC cells. In addition, we demonstrated that the mitochondrial respiratory inhibitor metformin decreased Erk1/2 phosphorylation and induced apoptosis and cell cycle arrest in PDAC cells partially through decreased mitochondrial ATP generation. Our current study uncovered a mechanism in which HSP60 promotes cancer cell growth revealing a potential therapeutic strategy targeting mitochondrial respiration in PDAC.

## Results

### Mitochondrial QC protein Hsp60 modulates tumorigenicity in PDAC

**Fig. 1:**
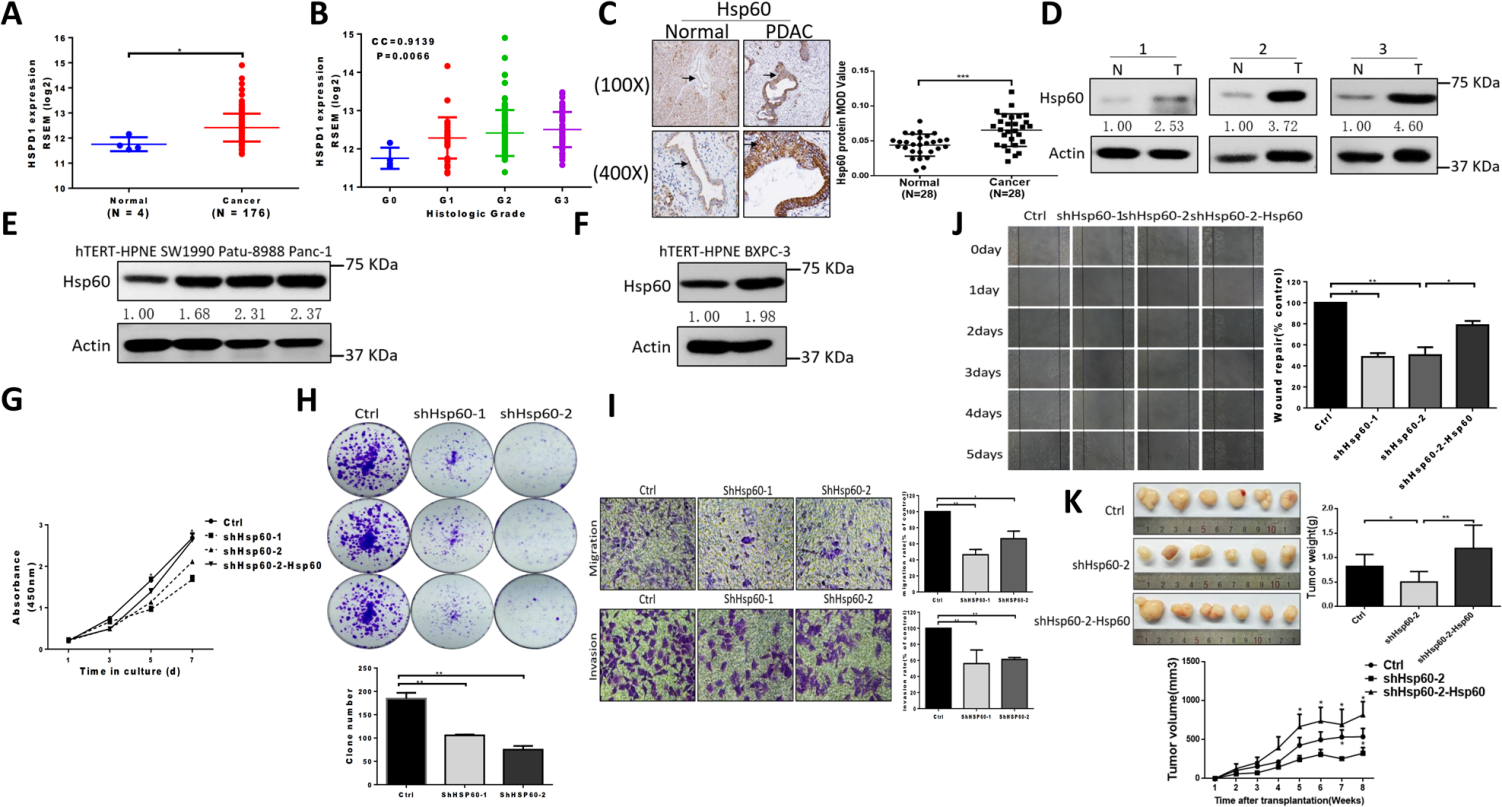
HSP60 modulates tumorigenicity in PDAC. **a** Analysis of relative HSPD1 expression in four normal pancreas tissues and 176 PDAC tissues from the TCGA database. **b** Normal and tumor samples were grouped by histological grade: G0 (*n* = 4), G1 (*n* = 31), G2 (*n* = 96), and G3 (*n* = 49). The column shows a dot map of HSPD1 expression in pancreatic cancer with different histological grades. CC correlation coefficient. **c** Representative photomicrographs of HSP60 immunohistochemistry (100× and 400×) of normal pancreas and cancerous tissues as well as quantitative immunohistochemistry results of its expression. **d** A representative western blot showing HSP60 protein expression in pancreas normal (N) and matched PDAC tissue (T). **e, f** Western blotting analysis of HSP60 protein expression in PDAC cell lines including cell lines **e** with and **f** without KRAS mutation. **g** Cell proliferation and **h** clone formation assays showing decreased proliferation in *HSP60* shRNA-transfected (shHSP60) Panc-1 cell lines compared with that found in control cells. **i** The migration (upper panel) and invasion (low panel) abilities of control and shHSP6060 Panc-1 cells were evaluated using a transwell assay. **j** The migration ability of control cells, shHSP60 Panc-1 cells, and rescued (ectopic) expression of *HSP60* cells was evaluated using a wound-healing assay. **k** Tumor xenograft experiments were carried out using shHSP60 Panc-1 cells compared with control cells and cells with rescued *HSP60* expression. Tumor volume and weight were calculated for each group (*n* = 6) at the indicated times after cancer cell injection, and statistical comparisons between the differences were assessed. All quantified data are presented as mean ± SEM (*n* ≥ 3). *P < 0.05, **P < 0.01, and ***P < 0.001

To investigate correlations between mitochondrial QC machinery and PDAC, we performed bioinformatics analysis in PDAC using the Cancer Genome Atlas (TCGA) database. Of the 19 most studied mitochondrial QC proteins (MQCPs), HSP60 (also known as HSPD1) was the only MQCP that had not only significantly increased expression in PDAC tissues (1.58-fold greater) compared with that of normal tissue, but was also positively correlated with PDAC histological grade (correlation coefficient = 0.91, *P* = 0.006) (Figs. 1a, b and Table 1). Consistently, protein analysis in both paraffinized PDAC and fresh tissue samples confirmed that HSP60 was elevated in cancerous tissues (Figs. 1c, d). Additionally, we found increased HSP60 in PDAC cell lines both with and without KRAS (a Kirsten ras oncogene homolog from the mammalian ras gene family) mutation (Figs. 1e, f, respectively) compared with that found in normal pancreatic ductal cells (hTERT-HPNE). As a related chaperone protein with HSP60, HSP10 (also known as HSPE1) was significantly increased in PDAC tissues compared with normal tissues with the same fold change observed with HSP60; however, expression of *HSP10* in PDAC tissues was not correlated with histological grade (Table 1). These findings indicate that *HSP60* expression is related to PDAC and that the relationship is independent of KRAS status.

**Table 1:** Expression of MQCP-related genes in PDAC compared with normal tissues in the TCGA database

**Gene name**	**Fold change** ^**a**^	***P-*** **value** ^**b**^	***P-*** **value** ^**c**^
	**(Cancer vs. normal)**	**(Cancer vs. normal)**	**(G0–G3)**
LONP1	0.9742	0.8781	0.0735
AFG3L2	1.2413	0.2016	0.1149
HSPA9	1.2761	0.0510	0.1999
CLPX	0.9306	0.6225	0.1569
CLPP	1.1323	0.4802	0.2417
PINK1	1.1234	0.4447	0.3430
HSPD1	1.5833	0.0183*	0.0066*
HSPE1	1.5965	0.0278*	0.1019
TRAP1	1.3614	0.0597	0.1277
HTRA2	0.8728	0.2238	0.3948
MEP1A	1.6511	0.5814	0.8060
MEP1B	2.0309	0.3788	0.1050
MIP	1.0208	0.9310	0.9999
PITRM1	1.0576	0.6482	0.7650
OSGEPL1	0.8253	0.1655	0.1356
YME1L1	1.0381	0.7323	0.3272
LACTB	0.8392	0.3188	0.0027*
PARK7	1.0203	0.8797	0.4806
SPG7	0.9244	0.5953	0.9232

^*^P-values ≤ 0.05 are considered significant

^a^ Fold change represents mRNAs differentially expressed in PDAC tissue compared with normal pancreas tissue

^b^ *P*-value (cancer vs. normal) indicating significantly different expression of mRNAs in PDAC tissue compared with normal pancreas tissue

^c^ *P*-value (G0–G3) indicating significantly different expression of mRNAs in pancreas and PDAC grouped by histological grade (G1, G2, and G3)

To characterize the impact of HSP60 on PDAC, we established two Panc-1 cell line models with stable knockdown (KD) of HSP60 at the level of 50% and 80%, respectively (Supplementary Figure S1A and B). Further, ectopic expression of *HSP60* in cells from one of the *HSP60* KD cell lines restored *HSP60* expression (Supplementary Figure S1C). We found that *HSP60* KD diminished in vitro PDAC cell proliferation and clonal formation, whereas rescued expression of *HSP60* increased the viability of cancer cells (Fig. 1g). Additionally, *HSP60* KD significantly reduced invasion (Fig. 1i, lower panel) and migration of cancer cells (Fig. 1i, upper panel, and Fig. 1j), whereas ectopic expression of *HSP60* in *HSP60* KD cancer cells reversed cancer cell migration (Fig. 1j). Furthermore, using an in vivo tumorigenicity assay, we found that both tumor weight (Fig. 1k, upper panel) and volume (Fig. 1k, lower panel) in mice with *HSP60* KD cells were significantly lower compared with control Panc-1 cells, whereas rescued expression of *HSP60* in *HSP60* KD cells increased tumor formation (Fig. 1k). Taken together, our findings indicate that *HSP60* expression modulates tumorigenicity, including cancer cell proliferation, invasion, and migration.

### *HSP60* maintains mitochondrial function in PDAC cells

Although remodeling of OXPHOS and glycolysis has been frequently observed in multiple cancer types, we analyzed the expression of genes involved in OXPHOS (83 genes), the TCA cycle (16 genes), and glycolysis (10 genes) in PDAC samples from the TCGA database (Supplementary Figure S2A), and we found that the expression levels of two glycolytic genes (*HK2* and *PKM2*), one TCA cycle gene (*IDH1*), and two OXPHOS-related genes (*COX6B2* and *NDUFAB1*) were positively correlated with histological grade score (Supplementary Figure S2B-F). Further, RNA levels of *COX6B2* and *NDUFAB1* were also significantly upregulated in PDAC tissues compared with normal pancreatic ductal tissues from the TCGA database (Supplementary Figure S2G and H). Although PDAC cells may have different metabolic profiles^9^, it is likely that OXPHOS function is increased, or at the least is not decreased, in PDAC cells compared with normal pancreatic cells.

**Fig. 2:**
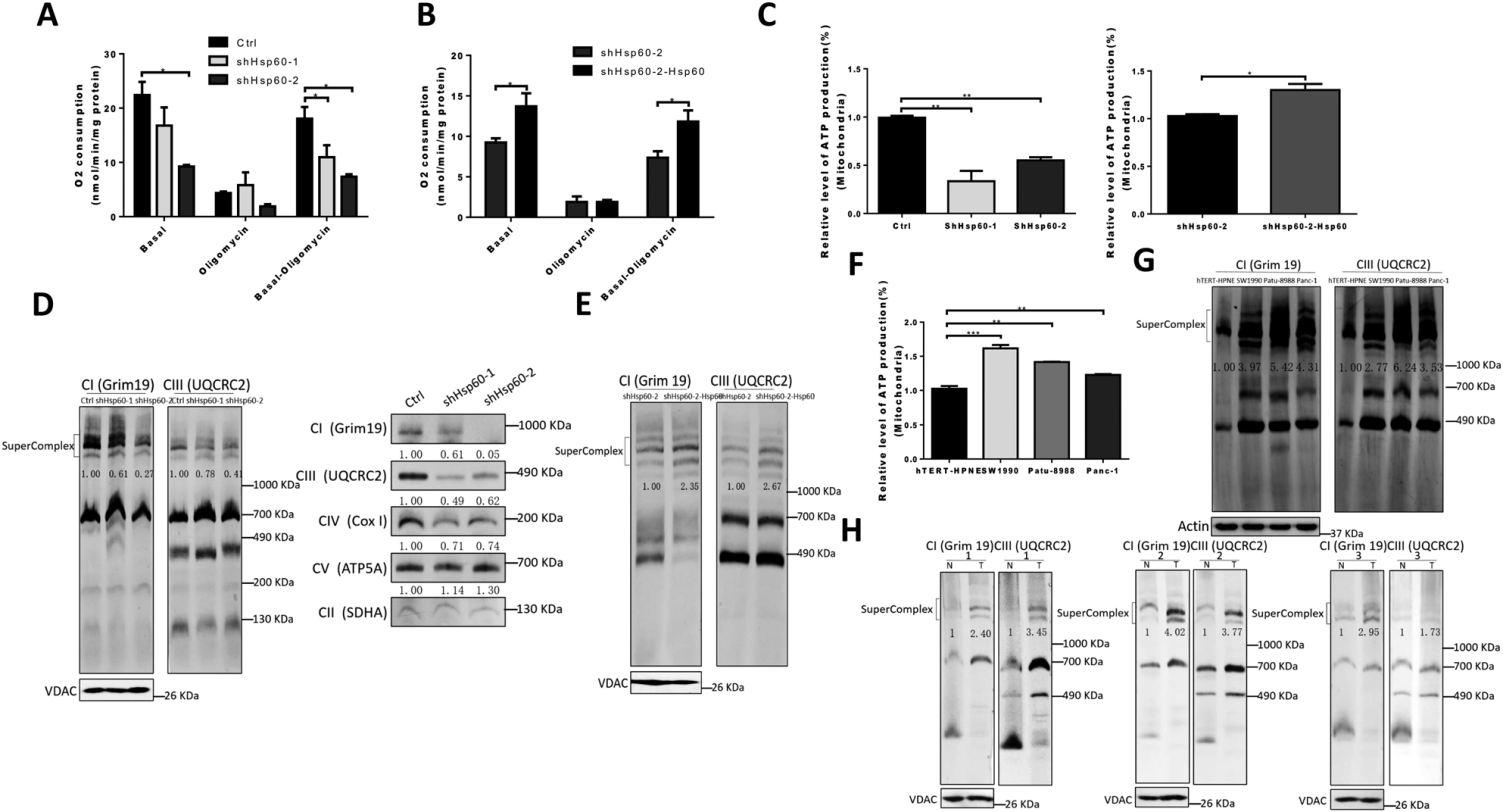
*HSP60* maintains mitochondrial function in PDAC cells. **a, b** Analysis of the oxygen consumption rate (OCR) of various cell lines by an Oxygraph-2k comparing shHSP60 Panc-1 cells with **a** control cells and **b** rescued expression of HSP60 cells. OCR was first measured of each cell line under basal conditions followed by sequential addition of oligomycin (100 μg/mL) at the indicated ATP-linked OCR. Basal, basal OCR; Oligomycin, OCR in the presence of 100 μg/mL oligomycin; Basal-oligomycin, OXPHOS-associated OCR was calculated by subtracting the OCR in the presence of oligomycin from basal OCR. **c** Measurements of mitochondrial ATP levels. Cells were incubated with 10 mM glucose or 5 mM 2-DG plus 5 mM pyruvate to determine mitochondrial ATP generation. Relative average ATP levels in mitochondria per cell line are shown. **d** Respiratory chain supercomplexes and single complexes in shHSP60 Panc-1 cells and control cells. **e** The respiratory chain supercomplex in shHSP60 Panc-1 cells and cells with rescued expression of *HSP60*. **f** Mitochondrial ATP levels and **g** respiratory chain supercomplexes were measured in cells from PDAC cell lines (SW1990, Patu-8988, and Panc-1) compared with normal pancreatic ductal cells (hTERT-HPNE). **h** Respiratory chain supercomplexes in fresh PDAC tissue samples compared with those found in paired normal tissues. The values of OCR and ATP generation were normalized to protein concentration. Blots were probed with anti-Grim19, anti-UQCRC2, anti-SDHA or anti-COX1, and anti-cyt c for complex I (CI), complex II (CII), complex III (CIII), and complex IV (CIV), respectively. All data are presented as mean ± SEM (*n* ≥ 3). *P < 0.05, **P < 0.01, and ***P < 0.001

Considering the important role of *HSP60* in the regulation of mitochondrial function and that OXPHOS may play a role in PDAC, we tested the effect of *HSP60* on OXPHOS in PDAC cells. First, we evaluated mitochondrial function by measuring mitochondrial respiration and ATP content in *HSP60* KD cells, and found that all basal mitochondrial respiration, uncoupled mitochondrial respiration, and coupled mitochondrial respiration were significantly decreased compared with control cells (Fig. 2a). In contrast, expression of ectopic *HSP60* in *HSP60* KD cells partially restored mitochondrial respiration (Fig. 2b). Furthermore, KD of *HSP60* decreased mitochondrial ATP content, whereas rescued expression of *HSP60* increased mitochondrial ATP content (Fig. 2c).

HSP60 is a chaperone protein indispensable for mitochondrial respiratory chain complex assembly. Thus, we next asked if *HSP60* KD downregulated the steady-state level of the mitochondrial respirasome in PDAC cells. As shown in the left panel of Fig. 2d, mitochondrial supercomplexes decreased by 40% and 70% in Panc-1 cells with of 50% and 80% of *HSP60* KD, respectively. Consistently, cells with *HSP60* KD exhibited decreased single mitochondrial respiratory chain complex I, III, and IV (Fig. 2d, right panel). In contrast, rescued expression of *HSP60* restored the steady-state level of mitochondrial supercomplexes (Fig. 2e). Similarly, PDAC cells of SW1990, Patu-8988, and Panc-1, all of which express higher levels of *HSP60* compared with normal pancreatic ductal cells, generated more mitochondrial ATP and exhibited a greater number of mitochondrial supercomplexes compared with that found in cells from the normal pancreatic ductal cell hTERT-HPNE (Figs. 2f, g). Our findings related to increased HSP60 and respiratory chain supercomplexes were independently verified in three fresh PDAC tissue samples compared with paired normal tissues (Figs. 1d,2h). Taken together, these results suggest that *HSP60* regulates mitochondrial function in PDAC cells.

### HSP60 promotes the phosphorylation of Erk1/2 via upregulation of OXPHOS

**Fig. 3:**
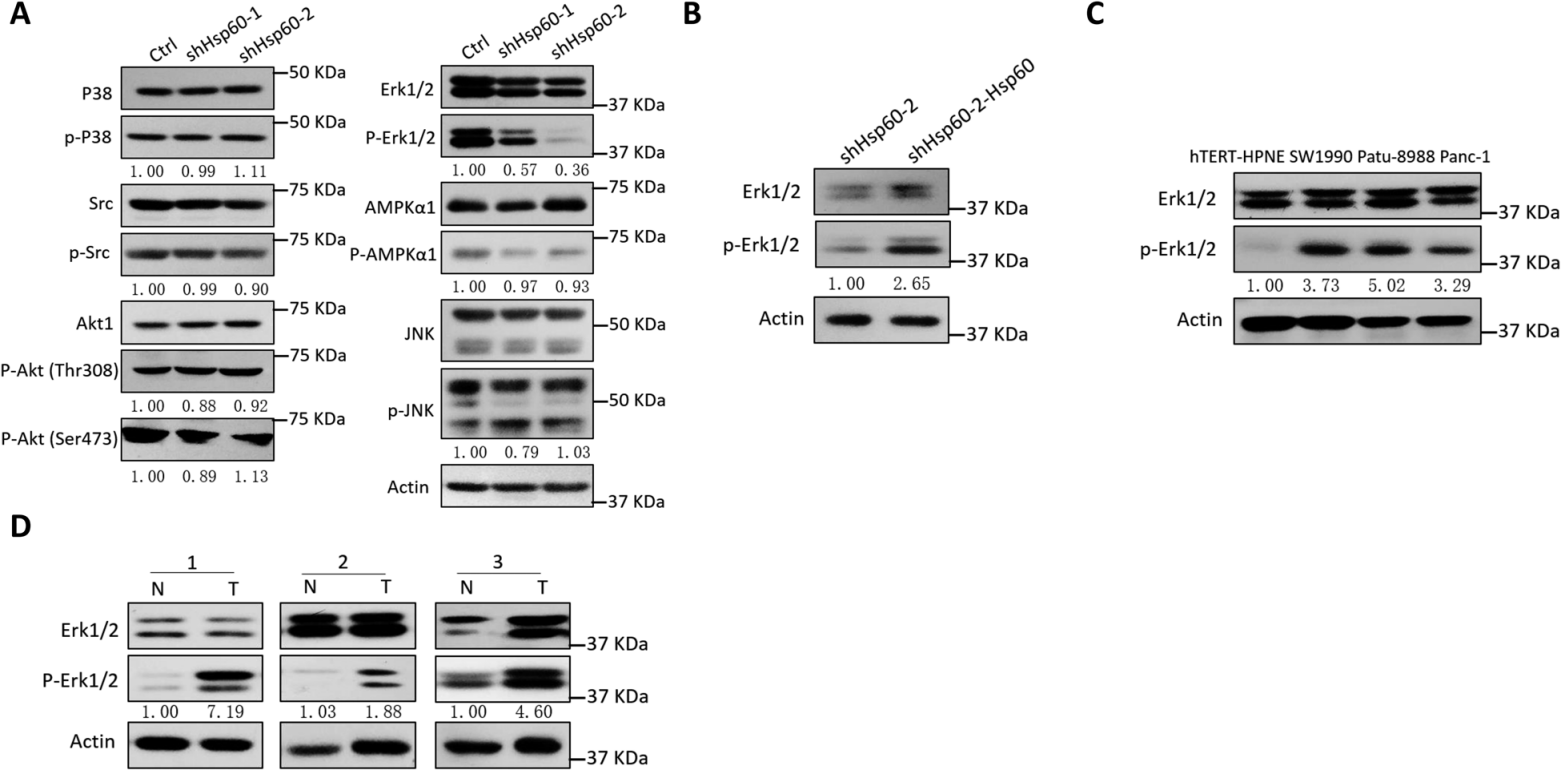
*HSP60* associated with the phosphorylation of Erk1/2. **a** Analysis of mitochondrial-to-nucleus pathways in shHSP60 Panc-1 cells and control (Ctrl) cells. Cell lysates were analyzed by western blot with antibodies against P38, p-p38, Src, p-Src, Akt1, p-Akt (Thr308), p-Akt (Ser473), Erk1/2, p-Erk1/2, AMPKα, p- AMPKα, JNK, p-JNK, and actin. **b–d** Western blot analysis of p-Erk1/2 in the indicated cells and tissues: **b** shHSP60 Panc-1 cells vs. cells with rescued expression of *HSP60*, **c** cells from PDAC cell lines (SW1990, Patu-8988, and Panc-1) vs. adjacent normal pancreatic ductal cells (hTERT-HPNE), and **d** fresh PDAC tissue vs. paired normal tissue. N adjacent normal tissue; T tumor tissue

As altered OXPHOS function has been linked to multiple mitochondrial-to-nucleus retrograde signaling pathways, we next investigated several key pathways involved in mitochondrial-to-nucleus signaling in cells from two *HSP60* KD Panc-1 cell lines and one *HSP60* KD cell line with rescued ectopic expression of *HSP60*. Of the six major mitochondrial-related pathways, decreased Erk1/2 phosphorylation was observed in *HSP60* KD cells compared with control cells (Fig. 3a), whereas Erk1/2 phosphorylation was increased in KD cells with rescued ectopic expression of *HSP60* (Fig. 3b). Consistently, we found that the increased HSP60 observed in KRAS wild-type and mutant PDAC cells compared with hTERT-HPNE cells (Fig. 1f) was also associated with increased phosphorylation of Erk1/2 compared with that of normal pancreatic ductal cells (Fig. 3c and Supplementary Figure S3). Similarly, fresh PDAC tissues, which exhibited increased levels of HSP60, also showed higher levels of phosphorylated Erk1/2 (Figs. 1d, 3d). These results show that *HSP60* overexpression in PDAC cells may be associated with Erk1/2 phosphorylation.

**Fig. 4:**
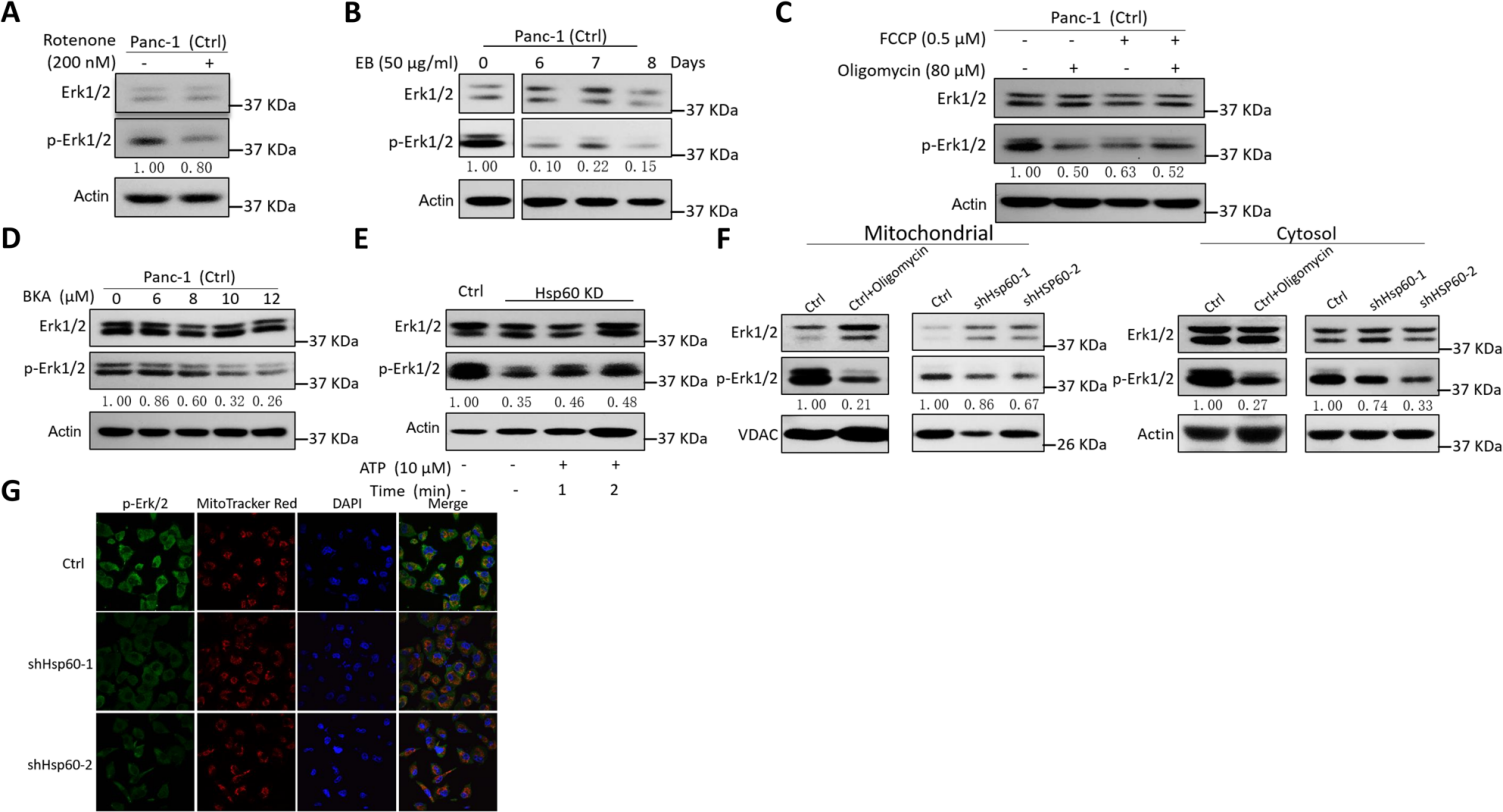
Decreased Erk1/2 phosphorylation in *HSP60* KD cells was mediated by attenuated OXPHOS function. **a–c** Inhibition of OXPHOS significantly affects the phosphorylation of Erk1/2. Western blot analysis of the effects of: **a** rotenone on the phosphorylation of Erk1/2 in cells treated with rotenone (200 nM) for 24 h, **b** Ethidium bromide (EB, an inhibitor of mitochondrial DNA replication) on the phosphorylation of Erk1/2 in cells treated with 50 μg/mL EB for 6–8 days, and **c** oligomycin on the phosphorylation of Erk1/2 in cells treated with 80 μM oligomycin for 24 h. **d, e** Mitochondrial ATP content significantly affects the phosphorylation of Erk1/2. Western blot analysis of the effects of **d**, Bongkrekic acid (BKA), an inhibitor of the adenine nucleotide translocator (ANT), on the phosphorylation of Erk1/2 in cells treated with BKA (0, 6, 8, 10, and 12 μM) for 24 h, and **e** ATP on the phosphorylation of Erk1/2 in shHSP60 Panc-1 cells treated with 10 mM ATP for 1 and 2 min. β-Actin was used as an internal control. **f** Western blot analysis of cytosol and mitochondrial p-Erk1/2 levels in Panc-1 control (Ctrl) cells treated with 80 µM oligomycin and shHSP60 Panc-1 cells compared with Ctrl cells. β-Actin and VDAC were used as cytosol and mitochondrial controls, respectively. **g** Colocalization of p-Erk1/2 to cytosol and mitochondria. Ctrl and shHSP60 Panc-1 cells were probed with p-Erk1/2 (green) and mitochondria (MitoTracker Red). All cells were background stained with DAPI (blue). The images were taken with a confocal laser microscope and representative images (600×) are shown

We next asked whether decreased Erk1/2 phosphorylation in *HSP60* KD cells was mediated by the attenuated OXPHOS function. As shown in Figs. 4a, b, inhibition of OXPHOS by either rotenone or Ethidium bromide (EB, inhibitor of mitochondrial DNA replication) reduced Erk1/2 phosphorylation in Panc-1 cells. To determine whether mitochondrial membrane potential (MMP) was involved in Erk1/2 phosphorylation, cells were treated with carbonyl cyanide-p-trifluoromethoxyphenylhydrazone (FCCP) and oligomycin to depolarize and hyperpolarize the MMP, respectively. Unexpectedly, we found that both FCCP and oligomycin treatment decreased Erk1/2 phosphorylation (Fig. 4c). Additionally, although *HSP60* KD caused oxidative stress in mitochondria (Supplementary Figure S4A), treatment using NAC in Panc-1 cells did not increase Erk1/2 phosphorylation in *HSP60* KD cells (Supplementary Figure S4B). Because *HSP60* KD impairs assembly of the respirasome followed by decreased mitochondrial ATP generation, we next determined whether the ATP generated from mitochondria is important for Erk1/2 phosphorylation. Antagonism of ATP translocation from mitochondria into the cytosol using bongkrekic acid (BKA), an inhibitor of the adenine nucleotide translocator (ANT), revealed that decreased ATP content in the cytosol inhibited Erk1/2 phosphorylation, a finding that indicates the ATP generated from OXPHOS is indispensable for Erk1/2 activation (Fig. 4d). Similarly, ATP complementation in *HSP60* KD cells partially restored Erk1/2 phosphorylation in a time-dependent manner (Fig. 4e).

As Erk1/2 is localized in both the outer mitochondrial membrane and the cytosol, we examined the status of Erk1/2 phosphorylation in both sites and found that inhibition of OXPHOS by oligomycin in Panc-1 cells decreased Erk1/2 phosphorylation in both mitochondria and cytosol (Fig. 4f). Similarly, *HSP60* KD also suppressed Erk1/2 phosphorylation in both mitochondria and cytosol (Figs. 4f, g). These results indicate that *HSP60* maintains OXPHOS function and promotes mitochondrial ATP generation, which in turn promotes Erk1/2 phosphorylation in PDAC cells.

### Metformin attenuates cancer cell growth via decreased mitochondrial ATP generation and subsequent Erk1/2 phosphorylation

**Fig. 5:**
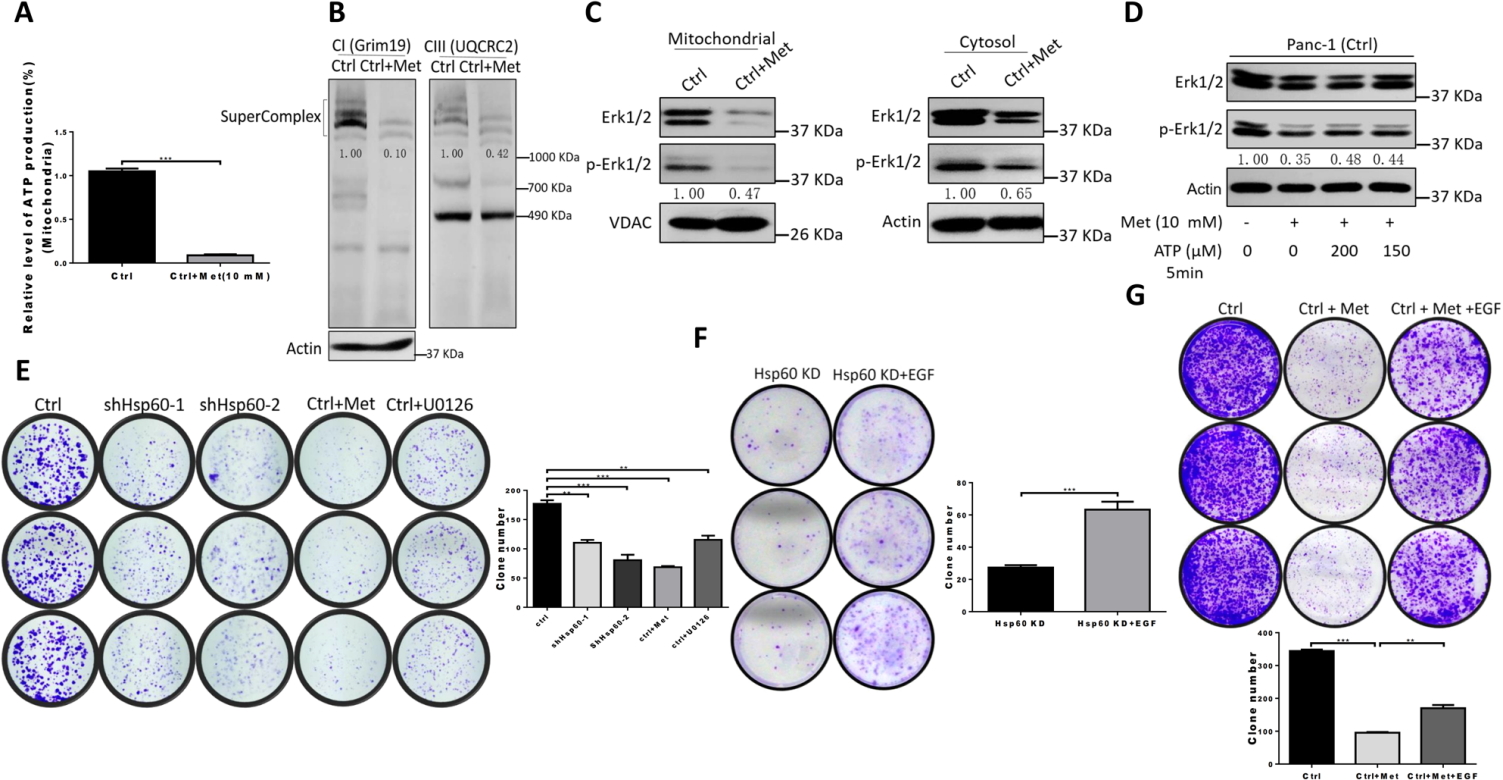
Metformin attenuates cancer cell growth via decreased mitochondrial ATP generation and subsequent Erk1/2 phosphorylation. **a** Mitochondrial ATP levels and **b** assembly of respiratory chain supercomplexes were determined in Panc-1 cells treated with 10 mM metformin (Met) for 72 h. **c** Western blot analysis of cytosol and mitochondrial p-Erk1/2 levels in Panc-1 (Ctrl) cells treated with 10 mM Met for 72 h. β-Actin and VDAC were used as cytosol and mitochondrial controls, respectively. **d** Western blot analysis of cytosol and mitochondrial p-Erk1/2 levels in Met pre-treated (72 h) Panc-1 (Ctrl) cells with and without ATP at concentrations of 150 nM and 200 nM for 5 min. **e** Clonal formation assay of Panc-1 Ctrl cells, *HSP60* KD cells, and Panc-1 Ctrl cells treated with 10 mM Met and 50 μM U0126 for 24 h. **f, g** Clonal formation assay of **f** *HSP60* KD and **g** Met-treated (10 mM) Panc-1 cells with or without EGF (30 ng/mL). U0126 is an inhibitor of the MAP kinase kinases, MEK1 and MEK2. Data are presented as mean ± SEM (*n* ≥ 3). **P < 0.01 and ***P < 0.001

Although the underlying mechanism is unclear, PDAC cell growth is inhibited by the anticancer drug candidate metformin. Because it has been demonstrated that metformin inhibits mitochondrial complex I activity^13^, we speculated that metformin may prevent Erk1/2 phosphorylation via decreased ATP generation to attenuate cancer cell growth. To test this hypothesis, we determined the level of the mitochondrial respirasome and confirmed that metformin impaired mitochondrial supercomplex assembly (Fig. 5a), through which mitochondrial ATP generation was dramatically decreased (Fig. 5b). Consistent with these findings, we also found that inhibition of mitochondrial ATP generation by metformin decreased Erk1/2 phosphorylation both in mitochondria and cytosol (Fig. 5c). In addition, ATP supplementation in cells treated with metformin partially rescued Erk1/2 phosphorylation (Fig. 5d). These results show that metformin inhibits Erk1/2 phosphorylation to some extent through downregulation of mitochondrial ATP generation. Furthermore, inhibiting Erk1/2 phosphorylation in control Panc-1 cells by U0126 (an inhibitor of the mitogen-activated protein (MAP) kinase kinases, MEK1 and MEK2) or metformin (Supplementary Figure S5 and Fig. 5c) mimics the effect of clonal formation found in *HSP60* KD cells compared with control Panc-1 cells (Fig. 5e). Moreover, treatment with EGF, which is an inducer of Erk1/2 phosphorylation, promoted clonal formation in both *HSP60* KD and metformin-treated Panc-1 cells (Figs. 5f, g), findings that support Erk1/2 phosphorylation has a role in *HSP60* KD-related and metformin-induced cancer cell growth inhibition. Of note, cells treated with metformin formed fewer clones than those cells incubated with U0126 (Fig. 5e). Furthermore, reactivated Erk1/2 did not fully restore clonal formation in Panc-1 cells treated with metformin (Fig. 5g). This finding indicates that the tumor inhibitory effects of metformin are partially mediated through regulation of Erk1/2 phosphorylation. Together, these findings indicate that decreased Erk1/2 phosphorylation in *HSP60* KD inhibits cancer cell growth and that OXPHOS inhibitors such as metformin may be used in the treatment of PDAC exhibiting increased Erk1/2 phosphorylation.

### *HSP60* KD and metformin administration induce apoptosis and cell cycle arrest in PDAC cells

**Fig. 6:**
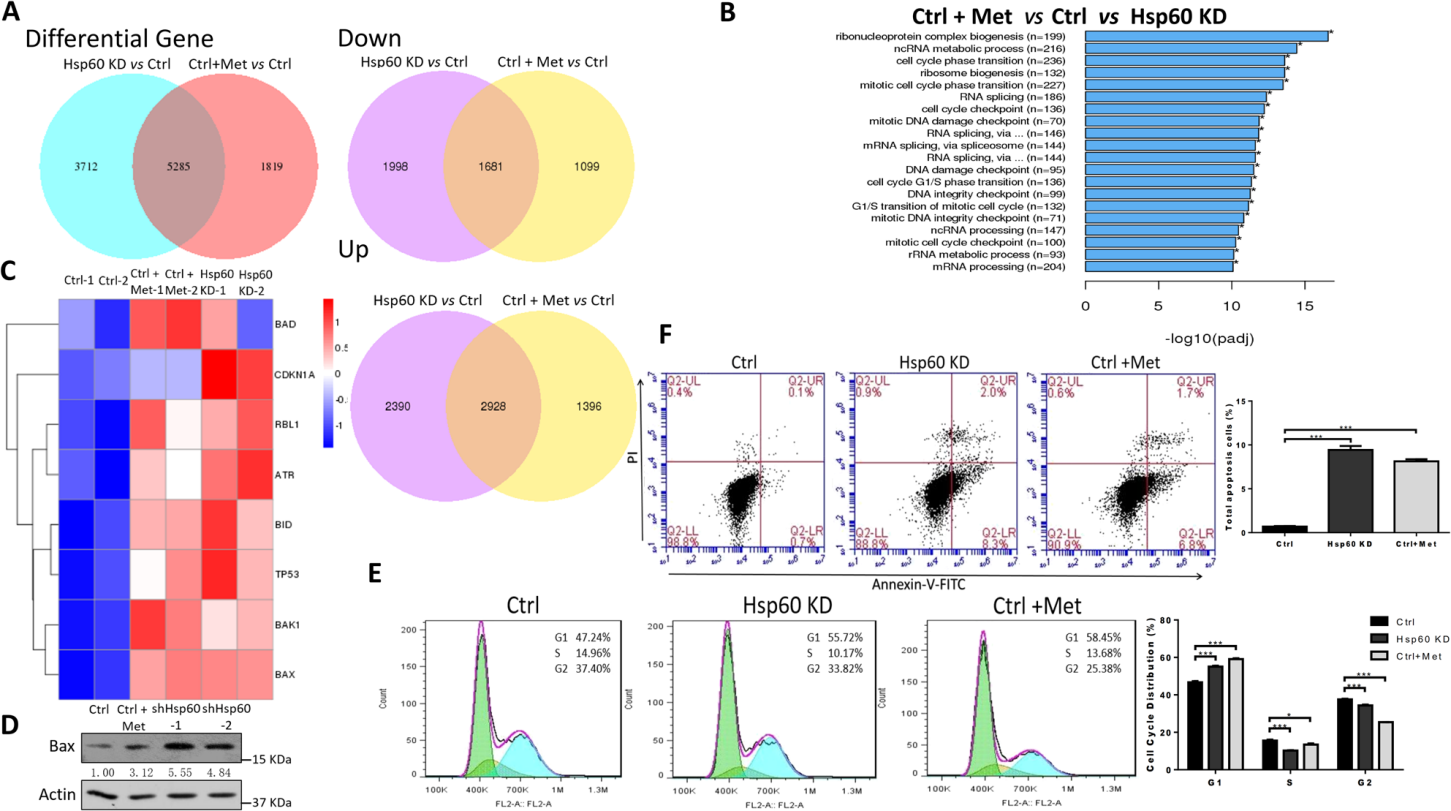
*HSP60* KD and metformin administration induce apoptosis and cell cycle arrest in PDAC cells. **a** DEGs were identified in *HSP60* KD and metformin-treated (10 mM for 72 h) Panc-1 cells. **b** Pathway enrichment analysis of 4,609 shared DEGs using the GO database. **c** Heat map showing hierarchical clustering of apoptosis and cell cycle genes related to p-Erk1/2 in *HSP60* KD and metformin-treated (10 mM for 72 h) Panc-1 cells. **d** A representative western blot showing HSP60 protein level in control Panc-1 cells, *HSP60* KD, and metformin-treated (10 mM for 72 h) Panc-1 cells. **e** Cell cycle stage and **f** apoptosis were determined by flow cytometry analysis in control Panc-1 cells, *HSP60* KD, and metformin-treated (10 mM for 72 h) Panc-1 cells. The column shows the percentages of cells at **d** different phases and **e** the percentage of apoptotic (early and late) cells. Data are presented as mean ± SEM (*n* ≥ 3). *P < 0.05 and ***P < 0.001

To gain further insights into the effects of *HSP60* KD and metformin on the inhibition of tumorigenesis, we next performed RNA-sequencing in wild-type Panc-1 cells, *HSP60*-silenced Panc-1 cells, and metformin-treated Panc-1 cells. We identified 8997 and 7104 differentially expressed genes (DEGs) in *HSP60*-silenced and metformin-treated PDAC cells, respectively (Fig. 6a). Of the 5285 common DEGs, we identified 4609 of which 1681 were downregulated and 2928 were upregulated in both *HSP60*-silenced and metformin-treated PDAC cells (Fig. 6a). Pathway enrichment analysis of the 4609 shared DEGs using Gene Ontology (GO) revealed a large proportion of significantly enriched pathways clustered into specific cell cycle-related processes, such as DNA replication, DNA repair, cell cycle check point, and ribosome biogenesis (Fig. 6b). Considering the fact that Erk1/2 plays an essential role in cell fate decisions, downstream effectors of Erk1/2 in mitochondrial apoptosis and the cell cycle were analyzed. As shown in Fig. 6c, we found drastically increased expression of mitochondrial apoptosis genes, including *BAX*, *BAK1*, and *BID*, in both *HSP60* KD and metformin-treated PDAC cells, of which protein level of BAX in control, *HSP60* KD, and metformin-treated PDAC cells was further confirmed (Fig. 6d). In addition, cell cycle negative regulators, such as *CDKN1A*, *RBL1*, *ATR*, and *TP53*, were upregulated (Fig. 6c). Consistently, *HSP60* KD and metformin treatment induced G1 cell cycle arrest and apoptosis in Panc-1 cells (Figs. 6e, f). These findings suggest that both metformin- and *HSP60* KD-induced PDAC cell growth inhibition is mediated by inhibition of Erk1/2 phosphorylation and activity of its downstream effectors.

## Discussion

To cope with the high protein turnover rate, QC proteins that monitor the folding of newly synthesized proteins and their subsequent assembly, as well as the disassembly of damaged proteins for degradation are over activated in multiple rapidly growing cancer cells^14^. However, activated chaperones such as *HSP90* are also indispensable to suppress cancer cell growth by stabilizing tumor suppressors, such as *P53* and *VHL*^15^. In mitochondria, QC proteins including heat shock family proteins and ATP-dependent proteases not only maintain mitochondrial proteostasis to protect the cells from stress induced apoptosis^16^, but also reprogram metabolism to facilitate cancer cell growth^17^. Of these QC proteins, *HSP60* is one the most studied chaperones in cancer biology^18^. Similar to *HSP90*, *HSP60* functions as either a tumor suppressor or promoter in different cancer types^5,19^. On the one hand, proteomics analysis identified HSP60 as a cancer-associated protein marker in both colorectal and breast cancers^20,21^, and in addition to mitochondrion, increased expression of *HSP60* was found in different subcellular structures^22^. On the other hand, *HSP60* may serve as a tumor suppressor in hepatocellular and clear cell renal cell carcinoma^5,23^. In PDAC, evaluation of *HSP60* expression in tumor was confounded by different detection methods^24,25^. In the current study, we demonstrated that *HSP60* expression was significantly increased in cancer tissues compared with that of adjacent normal tissues. Although we found no evidence of correlation between the histological grade and *HSP60* expression in our cohort of patients with PDAC, we found that *HSP60* expression was positively correlated with disease severity from the TCGA database. The reason for this discordant finding may be because of the limited local sample size. Considering that HSP60 and HSP10 are co-chaperones working together in mitochondria^26^, a coordinated expression pattern of these two proteins were frequently observed in different cancer types^27,28^ including PDAC (Table 1). Although more data are required to clarify the effects of *HSP60* expression on cancer progression, our study provides the first description of increased *HSP60* expression in PDAC.

As a key component of mitochondrial QC machinery, HSP60 guards mitochondrial function under multiple nutrition stress^3^. Constitutive expression of HSP60 in mitochondria is involved in stabilizing mitochondrial DNA(mtDNA) nucleoids and newly synthesized mtDNA encoded polypeptides^29,30^, indicating a pivotal role of HSP60 in the maintaining of OXPHOS function. In this study, we confirmed that HSP60 repression inhibited mitochondrial electron transfer and subsequent ATP synthesis, whereas elevated HSP60 boost the mitochondrial function. Generally, QC proteins including HSP60 ensure the proper mitochondrial protein folding to support the mitochondrial function at the protein level^31^, both loss/decreased of QC function and accumulation of misfolded mitochondrial protein that beyond the regulatory capacity of mitochondrial unfolded protein response, can activate organelle QC via mitophagy or mitochondrial fission and fusion^32^. Notably, HSP60 was shown to interact with RNA-binding protein FUS/TLS, and facilitate the mitochondrial fragmentation in addition to its QC property^33^. Although we did not look into the detail of mitochondrial fragmentation in this study, changes of mitochondrial mass was likely not affected in HSP60 KD cells (Fig. 4g). Additionally, HSP60 KD may change the mitochondrial cristae shape, which is important for mitochondrial supercomplex assembly^34^. Furthermore, HSP60 KD may also disturb the mitochondrial supercomplex assembly by impairment of newly synthesized OXPHOS subunits folding through HSP10/HSP60 complex^35^. However, further studies are required to clarify how HSP60 KD induced mitochondrial supercomplex assembly.

Although disturbances of mitochondrial function alter mitochondrial retrograde signaling^36^, altered expression of mitochondrial QC proteins was shown to boost cancer-related signaling pathways because of their pivotal roles in maintaining OXPHOS machinery^37^. *HSP60* activates many inflammatory related signaling pathways, but few of them are mitochondrial-related^38^. Recently, *HSP60* KD in renal cancer was shown to induce Reactive oxygen species (ROS) accumulation and subsequent activation of the adenosine monophosphate-activated protein kinase (AMPK) pathway. In our study, we found ROS accumulation in PDAC cells with *HSP60* KD, but AMPK was not significantly affected (Fig. 3a). Instead, we found that HSP60 was positively related with Erk1/2 phosphorylation via HSP60-induced cytokine secretion^39^. Our study reports for the first time that HSP60 is important for the activation of mitochondrial retrograde Erk1/2 phosphorylation. Previous studies have shown that *HSP60* functions as a tumor promoter by directly interacting with apoptosis/anti-apoptosis-related proteins to determine cancer cell fate. For example, HSP60 directly interacts with and inhibits xc of Bcl-xl, which is an anti-apoptotic mitochondrial outer membrane protein^40^. Further, it interacts with and stabilizes survivin to attenuate caspase-dependent apoptosis^41^, and forms a complex with clusterin to promote cancer cell survival and proliferation^42^. In this study, we uncovered a second mechanistic pathway of *HSP60*-regulated anti-apoptosis via an HSP60/OXPHOS/Erk1/2 axis.

Functional OXPHOS is indispensable to support cancer cell growth via selective activation of cancer-related mitochondrial retrograde signaling rather than energy supplementation^43^. Mitochondria contain multiple signal mediators, of which ATP is a sensor molecular for many pathways. Although Warburg effect is frequently observed in many cancer types, sustained OXPHOS function and subsequent ATP generation is vital in PDAC cells^11,44^. Our results showed that *HSP60* KD in PDAC cells reduce mitochondrial ATP generation and induces cell cycle arrest and apoptosis, whereas rescued ectopic expression of *HSP60* in *HSP60* KD cells re-boosts mitochondrial ATP generation and promotes cell proliferation. The effects of intracellular ATP generated from glycolysis on apoptosis were previously demonstrated^45^. In vitro supplementation of ATP was shown to promote Erk1/2 phosphorylation^46^. However, we show here that *HSP60* KD lowers endogenous mitochondrial ATP generation and activates mitochondrial apoptosis by reducing Erk1/2 phosphorylation in PDAC cells. Similarly, a causal relationship between mitochondrial ATP and ERK1/2 activation was also claimed in an ischemia/reperfusion model^47^. Furthermore, we confirmed that in vitro ATP supplementation promoted Erk1/2 phosphorylation in *HSP60* KD cells, whereas downregulation of OXPHOS function in PDAC cells with metformin decreased Erk1/2 phosphorylation and increased Erk1/2-related apoptosis. The effect of metformin on the inhibition of Erk1/2 phosphorylation has been proposed^48^; however, the underlying mechanism of metformin in the modulation of ERK1/2 is poorly understood. Under this context, we described in the present study an unconventional role of metformin in PDAC in which the administration of metformin caused overexpression of *HSP60*, increased activation of OXPHOS, and elevated Erk1/2 phosphorylation in PDAC cells. Although further studies are crucial to determine whether upstream effectors of Erk1/2 or Erk1/2 itself are regulated by mitochondrial ATP, OXPHOS inhibitors such as metformin may serve as potential drugs for the treatment of PDAC. In addition, further investigations are warranted to test the effectiveness of molecules such as metformin in PDAC cells with different levels of *HSP60* expression and OXPHOS activity.

In summary, our findings demonstrated for the first time that *HSP60* promotes cancer cell proliferation and serves as an anti-apoptotic mediator in PDAC. Investigation in the underlying mechanisms revealed that *HSP60* maintains OXPHOS function to generate ATP, which is essential for mitochondrial and cytosol Erk1/2 phosphorylation. Meaningfully, this improved understanding of the mechanism supports the use of anticancer agents such as metformin to target mitochondrial ATP generation in PDAC cells with high levels of *HSP60* expression, OXPHOS activity, and Erk1/2 phosphorylation.

## Materials and methods

### Cell lines and culture conditions

Pancreatic cancer cells SW1990 and BXPC-3 were ordered from Cell Resource Center, Chinese Academy of Medical Sciences in 2017. Pancreatic cancer cells Panc-1 and Patu-8988 were ordered from the same center in 2015. Test of the cells authentication were exempted for these cells from Cell Resource Center, Chinese Academy of Medical Sciences. Immortalized pancreatic ductal epithelium cells (hTERT-HPNE) was gifted by BaiRong Biotechnology (Shanghai, China) in 2017, and authenticated using short tandem repeat (STR) profiling analysis by the same center in August of 2017. All cells were cultured in high-glucose Dulbecco’s modified Eagle's medium (DMEM) (Thermo Fisher Scientific, Waltham, MA, USA) containing 10% cosmic calf serum (Sigma-Aldrich, St. Louis, MO, USA).

### Patients and PDAC tissue specimens

Twenty-eight pairs of 5-μm-thick formalin-fixed paraffin-embedded sections of primary PDAC specimens and paired adjacent normal pancreas tissues were obtained from patients at the First Affiliated Hospital of Wenzhou Medical University (Zhejiang, China). Three pairs of matched PDAC tumor tissues and adjacent normal tissues from each patient were immediately snap frozen in liquid nitrogen after surgical removal at the First Affiliated Hospital of Wenzhou Medical University. Informed consent was obtained from all subjects under protocols approved by the Ethical Committee of the First Affiliated Hospital of Wenzhou Medical University. All experimental methods were carried out in accordance with approved guidelines of Wenzhou Medical University.

### Immunohistochemistry

Tissue sections were mounted on slides, treated with xylene, and rehydrated using a graded series of alcohol–water solutions before heat treatment in 10 mM citric acid (pH 6.0) for antigen retrieval^49^. Endogenous peroxidase activity was quenched using a 0.3% H_2_O_2_ solution. After blocking with 10% serum, sections were incubated at 4 °C overnight with primary anti-HSP60 antibodies (sc-376261; 1:200; Santa Cruz Biotechnology, Dallas, TX, USA). The slides were then incubated with anti-mouse IgG antibody (ZSBIO, Beijing, China) at 37 °C for 30 min. Signals were visualized with diaminobenzidine (DAB) (ZSBIO), counterstained with hematoxylin, dehydrated in ethanol, cleared in xylene, and mounted. Protein expression levels were analyzed by calculating the integrated optical density per stained area (IOD/area, mean OD value, MOD) using Image-Pro Plus 6.0 (Media Cybernetics, Rockville, MD, USA) as described previously^50^.

### Antibodies and immunoblotting

Proteins from whole-cell and clinical tissues were extracted with RIPA lysis buffer (Cell Signaling Technology, Danvers, MA, USA) supplemented with a protease inhibitor cocktail (Sigma-Aldrich). Cytoplasmic and mitochondrial fractions were isolated from cultured cells as described previously^51^. Mitochondrial membrane proteins were extracted using digitonin or Triton X-100 (Sigma-Aldrich) as described in our previous study^52^. Proteins separated by blue native polyacrylamide gel electrophoresis (BN-PAGE) or sodium dodecyl sulfate polyacrylamide gel electrophoresis(SDS-PAGE) were transferred to 0.22 µm polyvinylidene difluoride (PVDF) membranes (Bio-Rad, Hercules, CA, USA) using a semi-dry transfer system (Bio-Rad). Proteins were probed with anti-Hsp60 (sc-376261; 1:1000; Santa Cruz Biotechnology), anti-ERK1/2 (#9102; 1:1000; Cell Signaling Technology), anti-phospho-ERK (Thr202/Tyr204) (#9101; 1:1000; Cell Signaling Technology), anti-AMPKα (#2532; 1:1000; Cell Signaling Technology), anti-phospho-AMPKα (Thr172) (#2535; 1:1000; Cell Signaling Technology), anti-P38 (#9212; 1:1000; Cell Signaling Technology), anti-phospho-P38 (Thr389) (#9211; 1:1000; Cell Signaling Technology), anti-Src (#2109; 1:1000; Cell Signaling Technology), anti-phospho-Src (#2105; 1:1000; Cell Signaling Technology), anti-AKT1 (#2938; 1:1000; Cell Signaling Technology), anti-phospho-AKT (Ser473) (#12694; 1:1000; Cell Signaling Technology), anti-phospho-AKT (Thr308) (#5106; 1:1000; Cell Signaling Technology), anti-JNK (#9252; 1:1000; Cell Signaling Technology), anti-phospho-JNK (#4668; 1:1000; Cell Signaling Technology), anti-NDUFA13 (ab110240; 1:1000; Abcam, Cambridge, MA, USA), anti-SDHA (ab14715; 1:1000; Abcam), anti-core2 (MS304; 1:1000; Abcam), anti-COXI (MS404; 1:1000; Abcam), anti-ATP synthase subunit alpha (ab14748; 1:1000; Abcam), anti-β-actin (sc-47778; 1:5000; Santa Cruz Biotechnology) or anti-VDAC (#4661; 1:1000; Cell Signaling Technology) antibodies, and then incubated with a horseradish peroxidase-conjugated anti-rabbit/mouse IgG (#7074 / #7076; 1:2000; Cell Signaling Technology) secondary antibody. Signals were detected with Super Signal West Pico chemiluminescent substrate (Thermo Fisher Scientific). Integrated optical density quantification was performed using a Gel-Pro Analyzer 4.0 (Media Cybernetics).

### Plasmids

*HSP60* and control luciferase short hairpin RNA (shRNA) were cloned into the lentivirus plasmid pLKO.1. The shRNA sequences for *HSP60* were as follows: shHSP60-1, 5′-TTCAAGAGCAGGTACAATG-3′ (ORF region); shHSP60-2, 5′-CCATCAGTTACTGGTTTCAGT-3′ (3′-UTR region). The shRNA sequence of luciferase was previously described^53^. For HSP60 overexpression, a full-length HSP60 complementary DNA (cDNA) was synthesized and cloned into pcDNA3.1. Stable cell lines were selected using 800 μg/mL G418 or 4 μg/mL puromycin (Sigma-Aldrich).

### Chemicals and reagents

Rotenone, oligomycin, FCCP, EB, ATP, *N*-acetyl cysteine (NAC), 2-deoxy-d-glucose (2-DG), and pyruvate were purchased from Sigma-Aldrich. BKA and metformin were obtained from Santa Cruz Biotechnology (Dallas, TX, USA) and U0126 was obtained from SelleckChem (Houston, TX, USA).

### Cell proliferation assay

A cell proliferation assay was performed using Cell Counting Kit-8^®^ solution (Dojindo, Gaithersburg, MD, USA) according to the manufacturer’s protocol. Briefly, cells were seeded in wells at a concentration of 4 × 10^3^ cells/100 μL in 96-well plates and treated with 10 μL of Cell Counting Kit-8^®^ solution during the last 4 h of culture. Optical density of the wells was measured at 450 nm using a Varioskan™ Flash Multimode Reader (Thermo Fisher Scientific).

### Colony formation assay

For colony formation assays, cells were seeded in six-well plates at a density of 1 × 10^3^ cells per well. After 13–15 days, colonies were fixed with 4% paraformaldehyde and stained with crystal violet. Cell colonies whose diameters exceeded 0.5 mm were counted using Gel-Pro Analyzer 4.0 (Media Cybernetics).

### Cell migration and motility analysis

Cells were cultured in six-well plates incubated overnight yielding confluent monolayers for the wound-healing test. Wounds were made using a pipette tip and photographs were taken immediately (time zero) and once daily until wound healing. The distance migrated by the cell monolayer to close the wounded area during this time period was measured. The migration of cells into the wound area was quantified by Image J v 2.4.1.7 (National Institutes of Health, Bethesda, MD, USA).

The cell motility assay was performed using a 24-well transwell plate chamber with a pore size of 8 μm (Costar, Cambridge, MA, USA). Cells (2 × 10^4^) were seeded in serum-free media and translocated toward complete growth media. After 24 h of incubation at 37 °C, the non-invaded cells on the upper membrane surface were removed with a cotton tip, and the cells that passed through the filter were fixed with 4% paraformaldehyde and stained with 0.1% crystal violet. The number of invaded cells was counted in three randomly selected high-power fields under a microscope.

### Matrigel invasion assay

The cell invasion assay was performed using a Transwell plate chamber. The inserts were coated with 20 μL Matrigel (1:9 dilution; BD Biosciences, San Jose, CA, USA). Cells (4 × 10^4^) were seeded in serum-free medium and translocated toward complete growth media. After 24 h of incubation at 37 °C, the remainder of the process was identical to that of the motility assay^54^.

### ATP measurement

ATP was measured using an ATP measurement kit (Molecular Probes, Carlsbad, CA, USA) according to the manufacturer’s instructions. To measure mitochondrial ATP, cells were incubated with 10 mM glucose (Sigma-Aldrich) or 5 mM 2-DG with 5 mM pyruvate for 2 h prior to measurement. Fluorescence/luminescence was measured using a Varioskan™ Flash Multimode Reader (Thermo Fisher Scientific).

### Measurements of oxygen consumption

Endogenous oxygen consumption from intact cells was determined using a Clark-type oxygen electrode (Oroboros Instruments, Innsbruck, Austria) as described previously^55^. After recording basal respiration, oligomycin (100 μg/mL) (Sigma-Aldrich) was added to measure un-coupling respiration.

### ROS measurement

Approximately 1 × 10^6^ cells were incubated with  6-chloromethyl-2',7'-dichlorodihydrofluorescein diacetate, acetyl ester (carboxy-H2DCFDA) (20 µm) (Thermo Fisher Scientific) for 30 min at 37 °C. Cells were washed and harvested in Hank’s buffered salt solution and fluorescence was record by a Varioskan™ Flash Multimode Reader (Thermo Fisher Scientific) with excitation at 488 nm and emission at 530 nm. Results are presented as the mean of fluorescence intensity.

### Immunofluorescence staining

Cells were cultured on coverslips and incubated in DMEM containing 100 nM MitoTracker Red (Invitrogen, Carlsbad, CA, USA) for 30 min at 37 °C in the dark. After staining, cells were washed with fresh growth medium, and fixed with 4% paraformaldehyde/phosphate-buffered saline (PBS) for 20 min. Cells were then permeabilized with PBS containing 0.2% Triton X-100 for 3 min at room temperature, and incubated overnight with anti-HSP60 (1:300; Santa Cruz Biotechnology) or anti-phospho-ERK (Thr202/Tyr204) (1:250; Cell Signaling Technology) antibodies at 4 °C in a darkroom. Next, cells were washed with PBS and incubated with a fluorescently labeled secondary antibody either IgG-Alexa Fluor 594 or IgG-Alexa Fluor 488 (1:300; Cell Signaling Technology) for 1 h at room temperature in the dark. A final incubation was performed to stain the cells with 4,6-diamidino-2-phenylindole (DAPI; Beyotime, Jiangsu, China) for 15 min at room temperature. After mounting the coverslips, images were captured using a confocal laser microscope at a magnification of ×600 (Nikon, Telford, UK).

### Apoptosis assay

Apoptosis assays were performed using an Annexin V- Fluorescein isothiocyanate (FITC) apoptosis detection kit according to the manufacturer’s protocol (Keygen Biotech, Jiangsu, China). Briefly, cells (1 × 10^6^) were collected and washed twice with PBS. Annexin V-FITC and propidium iodide (PI) (Keygen Biotech) were added and Fluorescence activated Cell Sorting (FACS) using a BD Accuri™ C6 (BD Biosciences, Ashland, OR, USA) was performed after 15 min of incubation at room temperature.

### Cell cycle analysis

Cells (1 × 10^6^) were collected, washed twice with PBS, and fixed with 75% cold ethanol at 4 °C for 12 h. After two washes with PBS, cells were co-incubated with 50 μg/mL propidium iodide (Keygen Biotech) and 10 μg/mL RNaseA (Keygen Biotech) at 4 °C for 30 min in the dark. Cells were then analyzed for DNA content by FACS using a BD Accuri™ C6 (BD Biosciences) and the results of the cell cycle were analyzed by FlowJo (Tree Star, Ashland, OR, USA).

### Xenograft experiments

All animal studies were conducted in accordance with the principles and procedures outlined in the institutional animal ethical committee of Wenzhou Medical University. Approximately 4 × 10^6^ Panc-1 cells were injected subcutaneously into 6-week-old female nude mice (Shanghai Laboratory Animal Center, Shanghai, China). Tumor volume (mm^3^) defined as (length × width^2^)/2 was measured using a caliper every 3–4 days up to study termination. After 8–9 weeks, all animals were sacrificed to measure the final tumor volume and tumor weight.

### Sample preparation and RNA sequencing

Total RNA was isolated from two biological replicates of each group of treated cells using the RNeasy Mini Extraction kit (Qiagen, Valencia, CA, USA). mRNA from 20 μg of total RNA was purified using polyT-attached magnetic beads followed by fragmentation. First- and second-strand cDNA were synthesized in library preparation for transcriptome sequencing. Clustering of index-coded samples was performed on a cBot Cluster Generation System using TruSeq PE Cluster Kit v3-cBot-HS (Illumina, San Diego, CA, USA) according to the manufacturer’s instructions. After cluster generation, the library preparations were sequenced on a HiSeq 2000 (Illumina).

### Sequencing annotation, identification, and pathway enrichment analysis of DEGs

Clean reads were obtained by deleting the adaptor-only sequences and low-quality sequences. Comparison of the sequences was carried out by BLASTN against the 1000 Genomes Build 37 Decoy 5. The number of annotated clean reads of each gene was analyzed and normalized as reads per kilobase per million reads. DEGs were identified as those genes with an adjusted *P* < 0.05 using DESeq2. GO enrichment analysis of DEGs was implemented by the clusterProfiler R package in which gene length bias was corrected. Pathway enrichment analysis based on GO analysis of DEGs was used to identify significantly enriched metabolic pathways or signal transduction pathways using an adjusted *P*-value < 0.05 as the threshold for significance.

### Statistical analysis

All experiments were performed in triplicate and were performed independently at least three times. Data are presented as the mean ± standard error of the mean (SEM). All statistical analyses were performed with SPSS 21.0 (IBM, Armonk, NY, USA). Significance was estimated using either independent Student’s *t*-test or one-way analysis of variance (ANOVA). A null hypothesis was rejected when *P < *0.05.

## Electronic supplementary material

Supplementary Figures
