# Supplementary material for: Oncogenic HSP60 regulates mitochondrial oxidative phosphorylation to support Erk1/2 activation during pancreatic cancer cell growth
Source: Cell Death Dis. 2018 Feb 7;9(2):161. doi: 10.1038/s41419-017-0196-z (PMC5833694; doi:10.1038/s41419-017-0196-z)

**Supplementary Figure S1.**

Validation of *HSP60* KD and *HSP60*-reexpression in Panc-1cell. **A, B,** *HSP60* KD in Panc-1 cells stably transfected with *HSP60* shRNA expression vector was confirmed by **A,** western blot and **B,** immunofluorescent staining (600×). **C,** Rescued expression of *HSP60* in shRNA-transfected Panc-1 cells (shHSP60-2).

**Supplementary Figure S2.**

Analysis of metabolism-related genes in normal pancreatic ductal tissue and PDAC tissues from the TCGA database. **A,** Expression of OXPHOS (83 genes), TCA cycle (16 genes), and glycolysis (10 genes) genes were analyzed in PDAC samples from the TCGA database. Samples were grouped by histological grade: G0 (n = 4), G1 (n = 31), G2 (n = 96), and G3 (n = 49). **B-F** Dot map showing altered expression of the following metabolism-related genes in pancreatic cancer with different histological grades: **B,** *HK2*, **C,** *PKM2*, **D,** *IDH1*, **E,** *NDUFAB1*, and **F,** *COX6B2*. Analysis of relative **G,** *COX6B2* and **H,** *NDUFAB1* expression in 4 normal pancreas tissues and 176 PDAC tissues from the TCGA database. Data are presented as mean ± SEM (n ≥ 3). CC, correlation coefficient.

**Supplementary Figure S3.**

Immunoblot of p-Erk1/2 in BXPC-3 cancer cells and normal pancreatic ductal cells.

**Supplementary Figure S4.**

Effect of ROS on Erk1/2 phosphorylation. **A,** Analysis of total ROS production in *HSP60* KD and control Panc-1 cells. **B,** Immunoblot of p-Erk1/2 in Panc-1 control (Ctrl) cells and *HSP60* KD Panc-1 cells treated with 200 μM NAC for 24 h. β-actin was used as an internal control.

**Supplementary Figure S5.**

Effect of U0126 on Erk1/2 phosphorylation. Immunoblot of p-Erk1/2 in Panc-1 control (Ctrl) cells treated with U0126 (30 μM, 40 μM, or 50 μM) for 24 h. β-actin was used as an internal control.


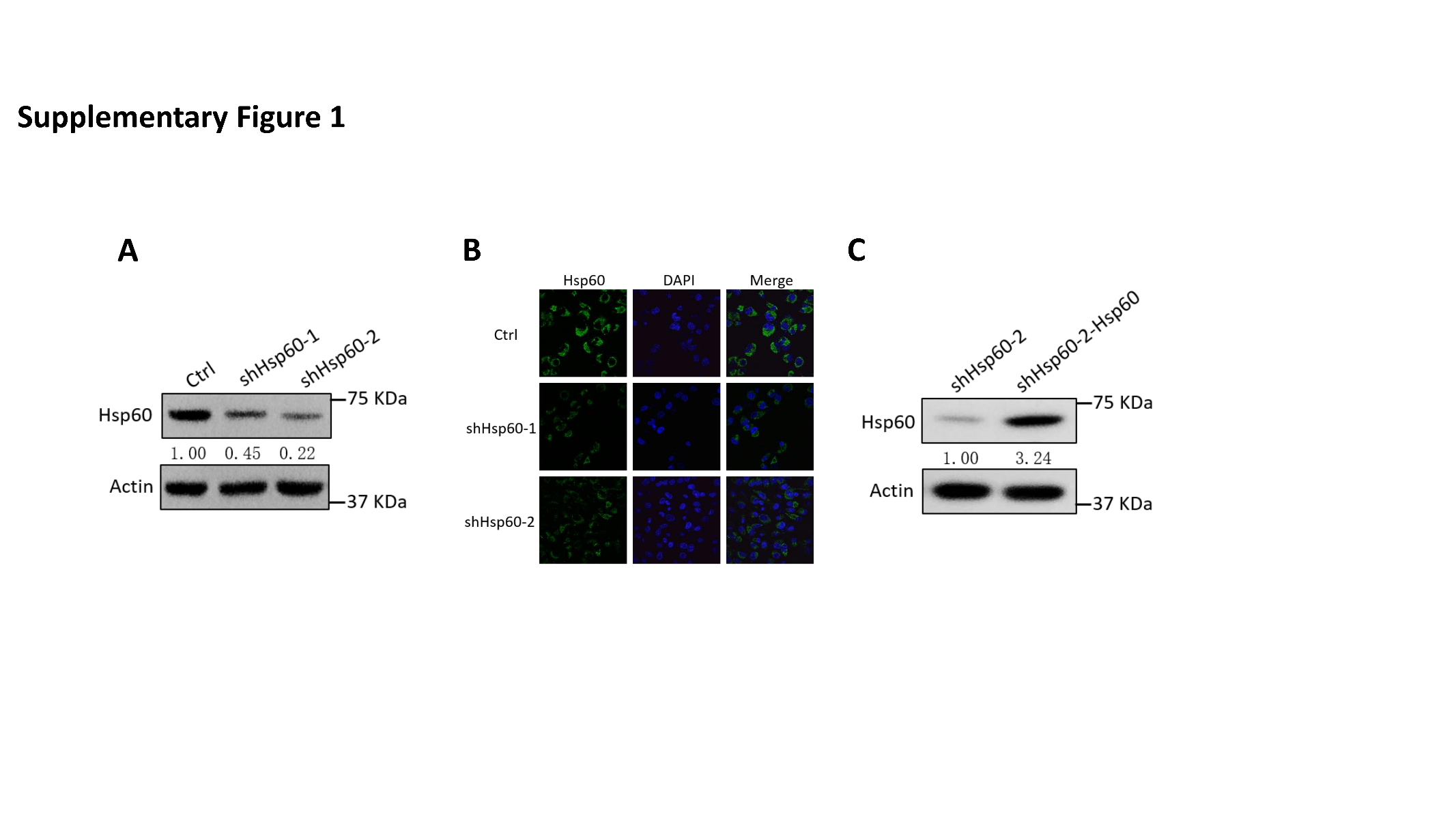


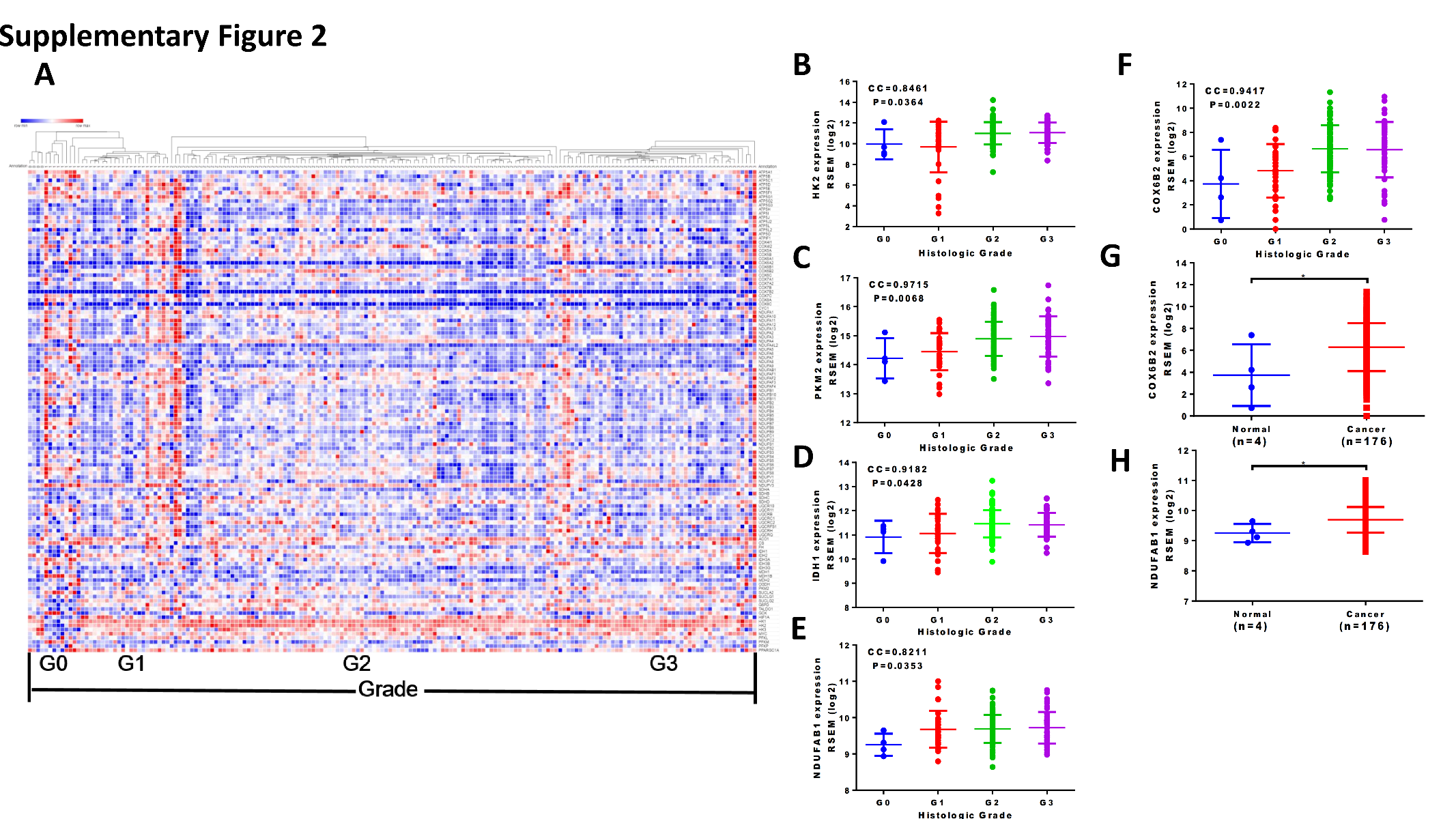


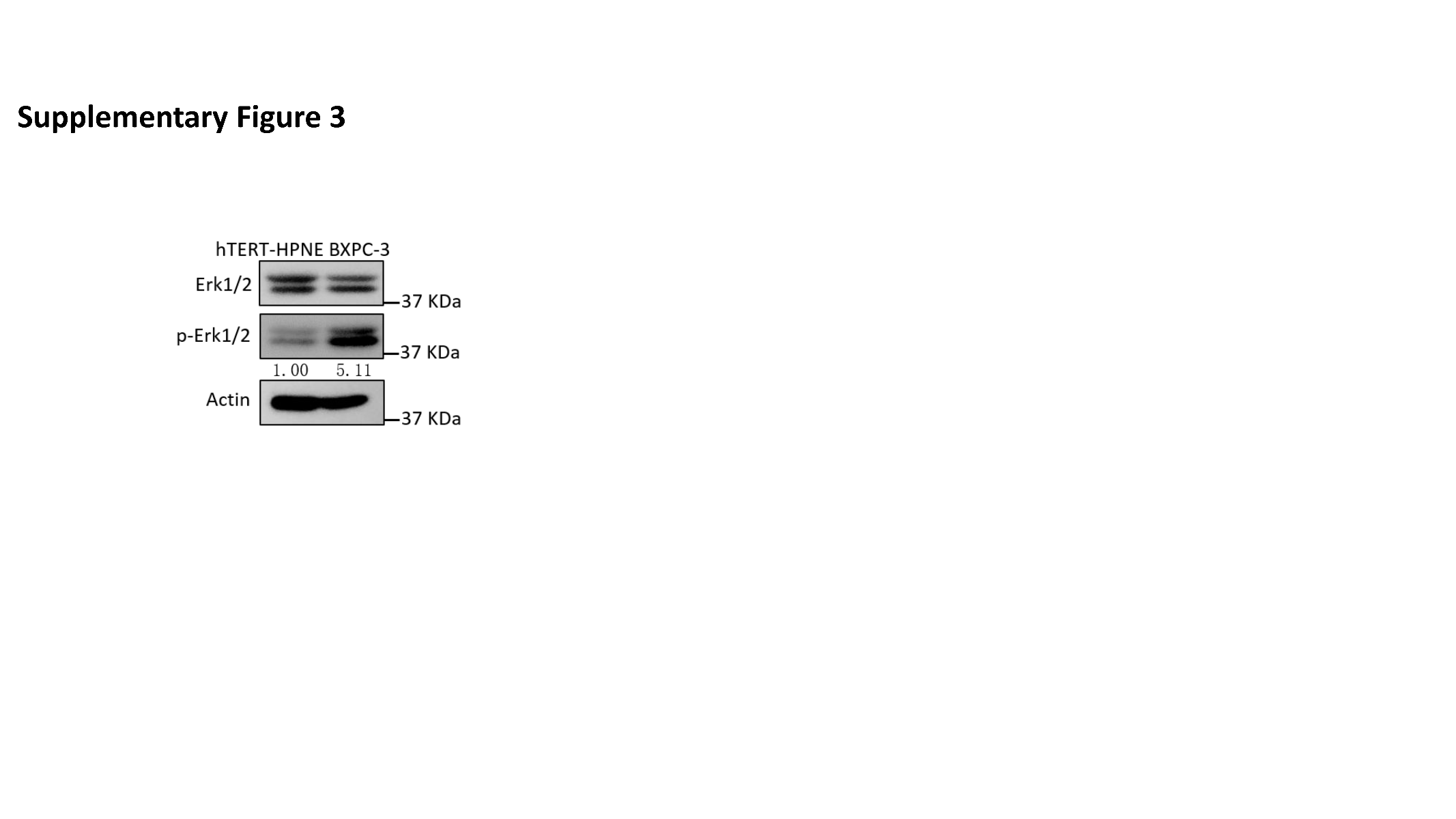


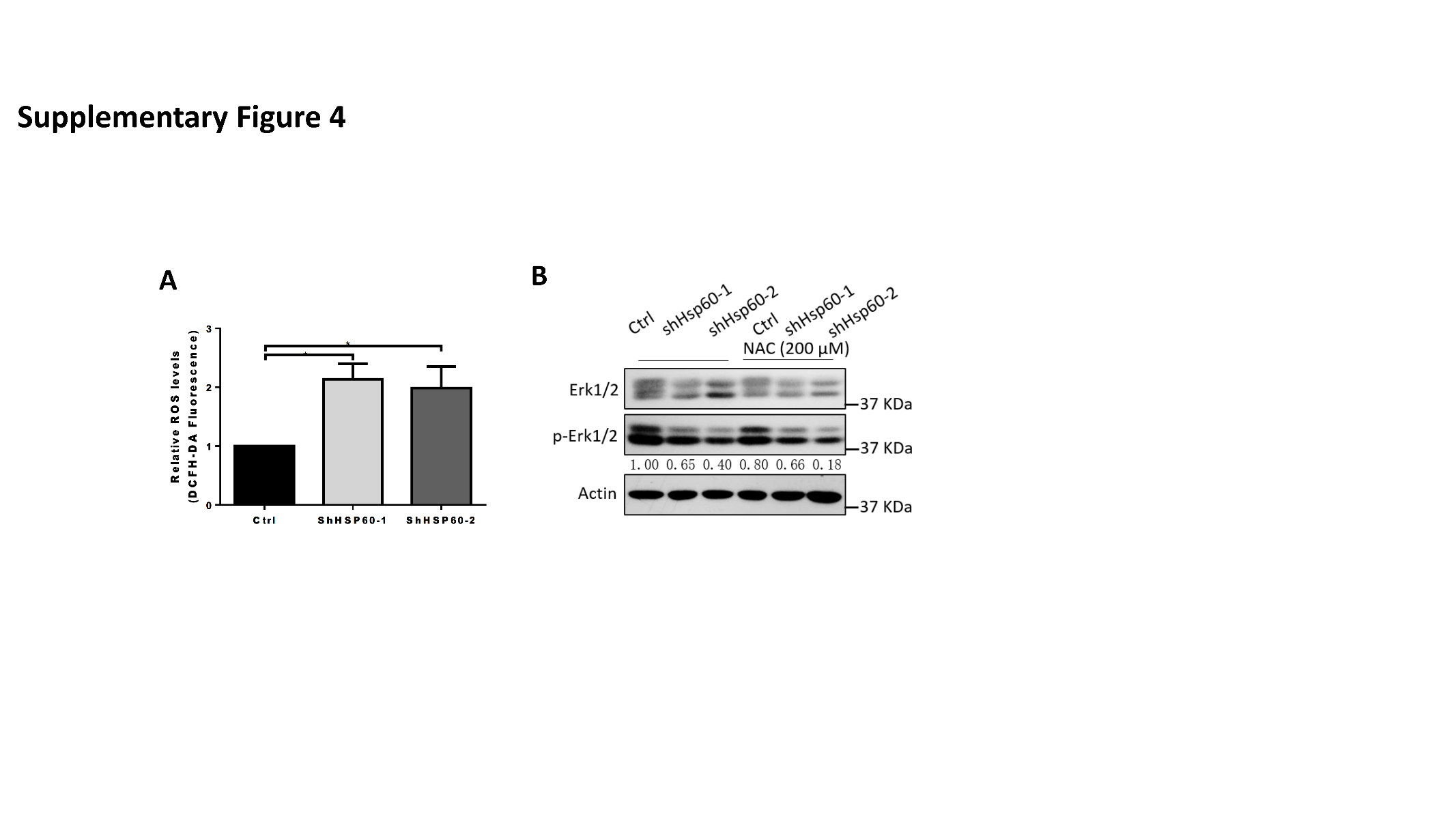


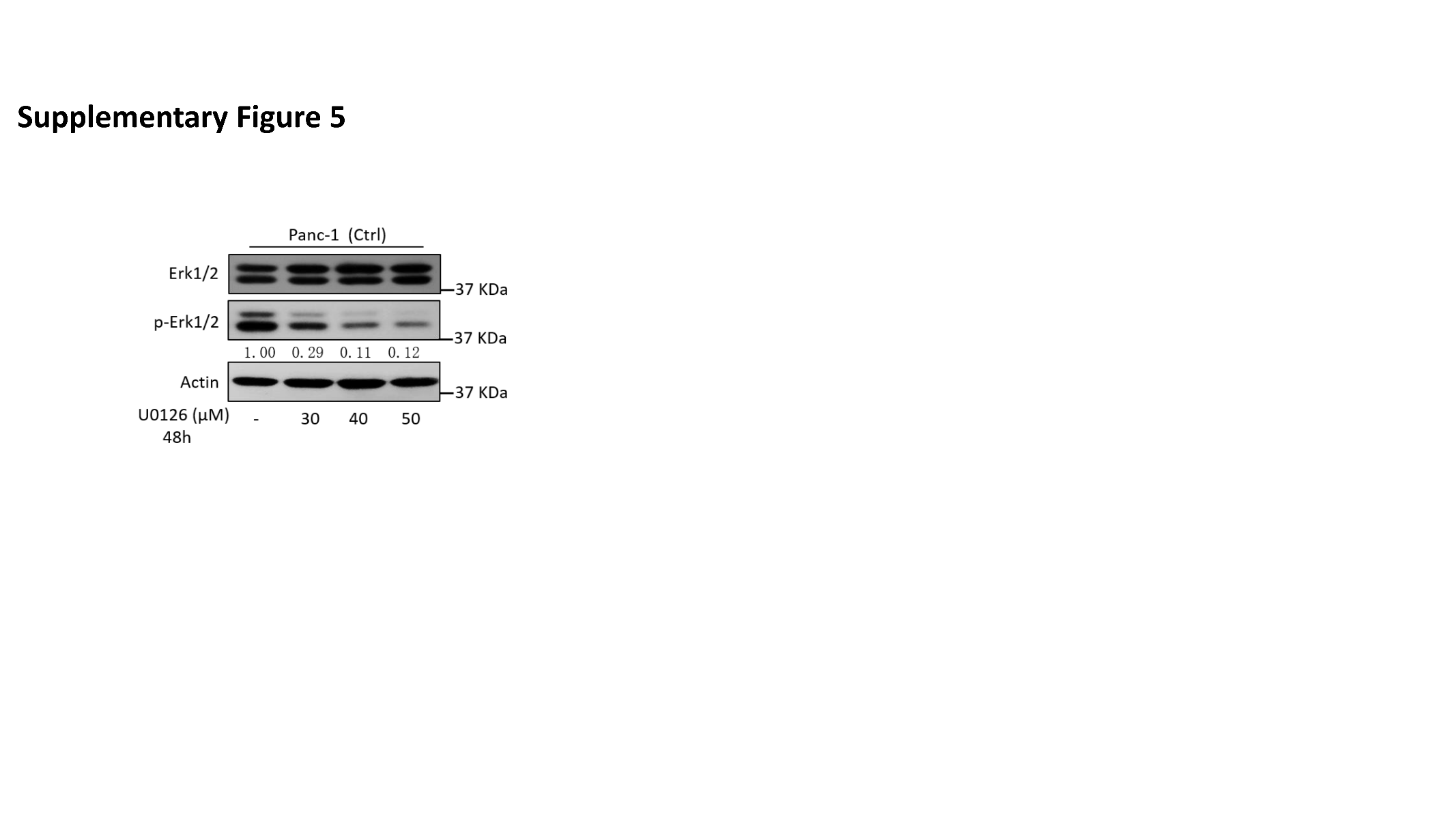

Supplement: Supplementary file 1 — Supplementary Figures [file 41419_2017_196_MOESM1_ESM.docx]
